# Risk factors for the development of postpartum depression in individuals who screened positive for antenatal depression

**DOI:** 10.1186/s12888-023-05030-1

**Published:** 2023-08-01

**Authors:** Jingjing Yu, Zhiyin Zhang, Yuanyuan Deng, Lijun Zhang, Chuncao He, Yinyin Wu, Xianrong Xu, Jun Yang

**Affiliations:** 1 Department of Nutrition and Toxicology, Hangzhou Normal University School of Public Health, 2318 Yuhangtang Rd, Hangzhou, 311121 Zhejiang China; 2 Department of Obstetrics, The Affiliated Hangzhou Women’s Hospital, Hangzhou Normal University, Hangzhou, China; 3 Department of Epidemiology and Biostatistics, Hangzhou Normal University School of Public Health, Hangzhou, China; 4 Zhejiang Provincial Research Center for the Diagnosis and Treatment of Uterine Cancer, The Affiliated Women’s Hospital, Zhejiang University, Hangzhou, China

**Keywords:** Antenatal depression, Postpartum depression, Sleep quality, Social support, Path analysis model

## Abstract

**Background:**

Women with antenatal depression often have a higher risk of developing postpartum depression (PPD) after delivery. A number of factors associated with the PDD in those previously reporting antenatal depression have been suggested, but further research is needed. This study aimed to investigate factors associated with developing subsequent postnatal depression in women who had screened positive for antenatal depression.

**Methods:**

This study was carried out in Hangzhou women’s Hospital. 578 women who experienced antenatal depression from this cohort were enrolled in this study. The sociodemographic and clinical characteristics of the participants were collected and tabulated against the incidence of postnatal depression. Binary logistic regression was used to estimate the effects of the principal underlying variables. The Chinese-version Edinburgh Postnatal Depression Scale (EPDS) was used to screen for PPD. Antenatal screening for depression was conducted at 28–34 weeks during pregnancy and postpartum depressive symptoms were assessed at 6 weeks after childbirth in the women. Path Analysis of Structural Equation Model (SEM) was employed to explore the direct, indirect, and total effects of risk factors of PPD.

**Results:**

57.6% (n = 333) of the participants subsequently developed PPD in our study. The results of the logistic analysis indicated that ages ≤ 35 years old (OR = 1.852; 95%CI: 1.002–3.423), non-one-child families (OR = 1.518; 95%CI: 1.047-2.200), and rare care from partner during pregnancy (OR = 2.801; 95%CI: 1.038–7.562), the antenatal EPDS score (OR = 1.128; 95%CI: 1.052–1.209), pyrexia during pregnancy (OR = 2.43; 95%CI: 1.358–4.345), fairly good (OR = 1.836; 95%CI: 1.009–3.340), fairly bad (OR = 3.919; 95%CI:2.072–7.414) and very bad postpartum sleep quality (OR = 9.18; 95%CI: 2.335–36.241) were associated with increased risk of PPD (compared to very good postpartum sleep quality). In path analysis model, antenatal EPDS score (standardized total β = 0.173) and pyrexia during pregnancy (standardized total β = 0.132) had both direct and indirect effects (the impact on outcome variables needs to be determined through other variables) on PPD. Sleep quality after delivery (standardized β = 0.226) and one-child family (standardized β = 0.088) had direct effects only on PPD.

**Conclusion:**

The results from our study indicated that more than 50% of the women who experienced antepartum depression would subsequently develop PPD. Depressive symptoms and pyrexia during pregnancy increase PPD scores, and these effects were in part mediated via poor sleep quality during the postpartum period.

**Supplementary Information:**

The online version contains supplementary material available at 10.1186/s12888-023-05030-1.

## Introduction

For most women, pregnancy is a momentous life event accompanied by marked psychological and physiological changes, which increase their vulnerability to the onset or recurrence of mental disorders [1]. Among these disorders, antenatal depression constitutes a major health challenge for pregnant women [2, 3]. Antenatal depression profoundly affects a woman’s sense of well-being, relationships, and quality of life, leading to adverse consequences such as difficulty performing common activities and failure to obtain prenatal care, the use of tobacco, alcohol, and other harmful substances, and the risk of self-harm or suicide [4, 5]. Antenatal depression may also affect fetal growth as well as infant temperament and later behavior in childhood [6, 7]. Moreover, antenatal depression is one of the strongest risk factors for postpartum depression (PPD), a condition linked to developmental problems in children [8]. Evidence from longitudinal studies indicated that, on average, 39% of women who experienced antenatal depression went on to have PPD, and 47% of those with PPD had also experienced antenatal depression [9]. The antenatal depression continuing to develop into postnatal depression not only lengthens the duration of depressive episodes in the mother, but significantly increases the risks of marital conflict and impaired infant-caregiver attachment, as well as the risks of impaired emotional, social, and cognitive development in the child [6, 10]. Therefore, developing strategies that prevent antenatal depression continuing to develop into PPD is of great importance for the health of both women and their offspring.

From the view of prevention, identifying modifiable risk factors is the first step toward primary prevention. Several studies have been conducted to investigate the associations between antenatal depression and PPD [11–15]. The results from these studies consistently revealed that antenatal depression was associated with significantly higher odds of PPD in women, with an odds ratio ranging from 1.50 [11] to 7.52 [12]. However, to the best of our knowledge, only Redshaw et al. investigated the risk factors for developing subsequent postnatal depression in mothers who experienced antenatal depression [16], and their results indicated that being left alone in labor and experiencing poor postnatal health was associated with increased risk of PPD in this population. Therefore, the factors that drive the continuous development of antenatal depression into PPD in women with established antenatal depression require further investigation. Moreover, as PPD is a complex disease that originates from the interactions between social, psychological, behavioral, and biological factors, the mechanisms underlying these interactions and their contributions to the continuous development of antenatal depression into PPD also require further assessment.

To address this issue, we conducted a nested case-control study to investigate the factors associated with the continuous development of antenatal depression into PPD in women with established antenatal depression. We enrolled women with new-onset antenatal depression and analyzed the characteristics of women with or without PPD at the end of the follow-up, trying to clarify the key factors that influence the outcomes of PPD who were screened positive for antenatal depression in this population. Potential risk factors were selected under the framework of stress process theory [17], which described the model of a health problem with three basic concepts: stressors, mediators, and outcomes. Previous studies have shown that sociodemographic characteristics of pregnant women can be important stressors of PPD, while their family support status and personal coping ability play mediating role during this process [18, 19]. Therefore, in this study, factors including socio-demographics, clinical features, family support, and individual coping ability were incorporated in the analysis. Furthermore, we use a path analysis of structural equation model to specify the underlying relationships among the factors that characterize the process through which women with antenatal depression continue to develop into PPD.

## Methods

### Participants and general procedure

Participants of this study were drawn from a cohort designed to explore the determinants of antenatal depression and postnatal depression in women, which was carried out in Hangzhou women’s Hospital. Pregnant women who met the inclusion-exclusion criteria were included in this study. Women were eligible for inclusion if they were between 28 and 34 weeks gestation. Women were excluded as follows: (1) women with 18 years old or older; (2) able to understand the content of the questionnaire; (3) without a history of anxiety and depression. This study was approved by the Ethics Committee of Hangzhou Women’s Hospital (No. 2020-01-06) and was carried out following the approved guidelines. Initially we contacted more than 6000 pregnant women during their hospital visit. However, many women refused to participate, either because they did not have enough time to listen to our presentations or complete our questionnaire, or had other concerns. Finally, a total of 2243 women were included in the cohort and 578 women with new-onset antenatal depression were enrolled in this study.

### Measures

Demographic data of the participants were collected from the medical records by trained staff. Basic information collected was spilt into (1) general characteristics (age, education level, family income, gravidity, history of abortion, family support, health status etc.); (2) previous or current disease status, sleep quality, perinatal complications, delivery experiences and lifestyle habits.

**Fig. 1:**
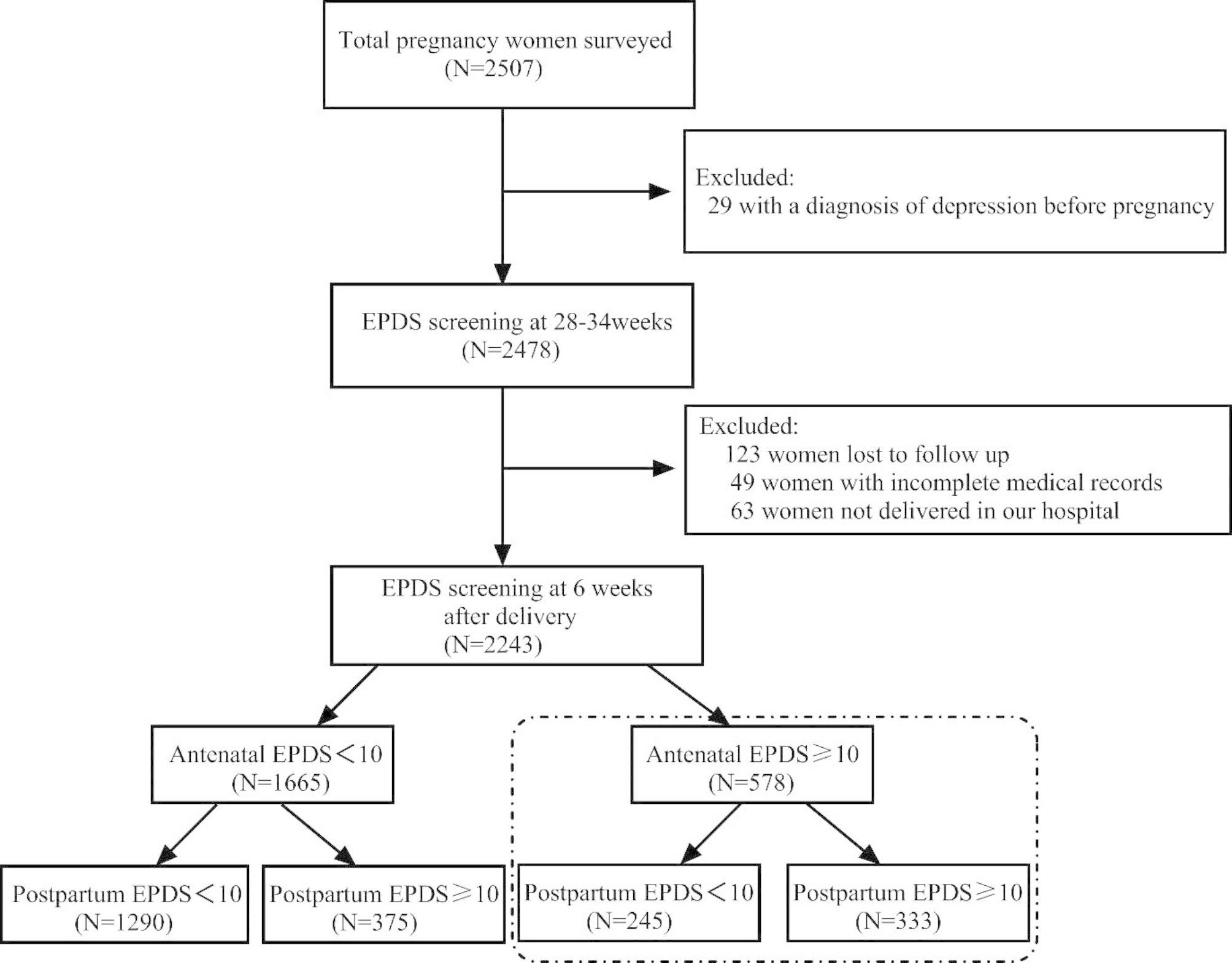
The flowchart of participants, from recruitment to final follow-up.

In this study, maternal depressive symptoms during pregnancy were assessed by the Chinese version of Edinburgh Postnatal Depression Scale (EPDS) [20], which has been demonstrated to have excellent psychometric properties in Chinese population [21]. The EPDS scale contains 10 items and total scores ranged from 0 to 30, with higher scores indicating more severe depression. According to the information from the reliability and validity study of the Chinese version of the EPDS, 10 was taken as the cutoff point for the detection of risk for clinical depressive symptoms [20, 22]. Antenatal screening for depression was conducted at 28–34 weeks during pregnancy and postpartum depressive symptoms were assessed at 6 weeks after childbirth in the women. The flow chart of our implementation process was shown in Fig. 1.

### Statistical analysis

In the current study, nested case-control design was applied focusing on those women who had experienced antenatal depression in the cohort. Sociodemographic and clinical characteristics were described and compared between women with or without PPD. This was followed by multiple logistic regression analysis to examine all potential risk factors associated with PPD and the results were visualized with the “forestplot” package in R 4.0 (R Core Team, 2017).

Path analysis of structural equation model (SEM) was constructed to explore the possible links between sociodemographic characteristics, lifestyle habits, and clinical characteristics with PPD in women. Six parameters, including the Sartorra-Bentler Chi-squared test of fit (*P* > 0.05), CMIN (chi-square, χ^2^), cardinality of freedom ratio (normal chi-square, χ^2^/*df*, CMIN/DF＜3.00 is acceptable), comparative fit index (CFI ≥ 0.90 is acceptable, ≥ 0.95 is good); adjusted goodness-of-fit index (AGFI ≥ 0.90 is acceptable, ≥ 0.95 is good); root mean square error of approximation (RMSEA ≤ 0.08 is recommended) were used to judge the degree of fit between the model and the data. The Akaike Information Criterion (AIC) and the Bayesian Information Criterion (BIC) were also calculated to evaluate the fit of the model, with lower values representing a better fit. Both direct and indirect effects of multiple interacting variables on PPD were presented. The Amos 24.0 (IBM SPSS Statistics 23) was used to build the SEMs.

## Results

### The sociodemographic and clinical characteristics of the participants

In our longitudinal study, a total of 2243 women had completed both rounds of depression screening, with 578 women diagnosed with antenatal depression. The sociodemographic and clinical characteristics of the participants were presented in Table 1. The age of the women ranged from 22 to 43 years (Mean: 30.2 years, SD: 3.3 years), and more than 90% of them were ≤ 35 years. 79.6% of the women had completed undergraduate degree, 13.0% had graduate degree, and the rest were high school educated or below. According to the information from the reliability and validity study of the Chinese version of the EPDS, 10 was taken as the cutoff point for the detection of risk for clinical depressive symptoms [20]. Based on this cutoff score, 57.6% of the original sample (n = 333) was found to have PDD. The results showed that there were significant differences in education level, one-child family, and the levels of spouse care during pregnancy between women with and without PPD.

**Table 1:** Sociodemographic and clinical characteristics of the participants

Characteristics	Overall (n = 578)	Postpartum depression		*P-*value	
		No(n = 245)	Yes (n = 333)		
**Sociodemographic characteristics**					
Maternal age (y), Mean ± SD/n(%)	30.24 ± 3.331	30.44 ± 3.384	30.08 ± 3.287		0.198
35	521 (90.1)	215 (87.8)	306 (91.9)		0.099
35	57 (9.9)	30 (12.2)	27 (8.1)		
Pre-pregnancy BMI (kg/m^2^), Mean ± SD/n (%)	20.75 ± 3.016	20.87 ± 2.914	20.66 ± 3.09		0.411
18.5	117 (20.2)	46 (18.8)	71 (21.3)		0.507
18.5–23.9	385 (66.6)	166 (67.8)	219 (65.8)		
24-27.9	59 (10.2)	28 (11.4)	31 (9.3)		
≥28	17 (2.9)	5 (2.0)	12 (3.6)		
Ethnicity, n (%)					
Han	567 (98.1)	241 (98.4)	326 (97.9)		0.683
Others	11 (1.9)	4 (1.6)	7 (2.1)		
Education level, n (%)					
Lower than high school	12 (2.1)	8 (3.3)	4 (1.2)		0.040*
High school	31 (5,4)	9 (3.7)	22 (6.6)		
College	460 (79.6)	189 (77.1)	271 (81.4)		
Higher than college	75 (13.0)	39 (15.9)	36 (10.8)		
Household yearly income (Ten thousand CNY), n(%)					
＜8	41 (7.1)	12 (4.9)	29 (8.7)		0.34
8–15	131 (22.7)	58 (23.7)	73 (21.9)		
15–30	227 (39.3)	100 (40.8)	127 (38.1)		
＞30	179 (31.0)	75 (30.6)	104 (31.2)		
One-child family, n(%)					
Yes	216 (37.4)	105 (42.9)	111 (33.3)		0.019*
No	362 (62.6)	140 (57.1)	222 (66.7)		
Pregnancy condition, n(%)					
Planned	377 (65.2)	167 (68.2)	210 (63.1)		0.203
Unplanned	201 (34.8)	78 (31.8)	123 (36.9)		
Primipara, n(%)					
Yes	446 (77.2)	182 (74.3)	264 (79.3 )		0.158
No	132 (22.8)	63 (25.7)	69 (20.7)		
Partner Support, n(%)					
Rarely	31 (5.4)	6 (2.4)	25 (7.5)		0.019*
Sometimes	164 (28.4)	67 (27.3)	97 (29.1)		
As usual	383 (66.3)	172 (70.2)	211 (63.4)		
Expose to tobacco before pregnancy, n(%)					
Yes	27 (4.7)	10 (4.1)	17 (5.1)		0.564
No	551 (95.3)	235 (95.9)	316 (94.9)		
Expose to alcohol before pregnancy, n(%)					
Yes	306 (52.9)	119 (48.6)	187 (56.2)		0.071
No	272 (47.1)	126 (51.4)	146 (43.8)		
**Clinical characteristics**					
EPDS score at visit 1, Mean ± SD	12.63 ± 2.97	12.00 ± 2.42	13.10 ± 3.205		< 0.001*
Abortion, n(%)					
0	401 (69.4)	171 (69.8)	230 (69.1)		0.851
1+	177 (30.6)	74 (30.2)	103 (30.9)		
Physiological reactions during pregnancy, n(%)					
Rarely	126 (21.8)	58 (23.7)	68 (20.4)		0.446
Sometimes	419 (72.5)	171 (69.8)	248 (74.5)		
Most of the time	33 (5.7)	16 (6.5)	17 (5.1)		
Pyrexia during pregnancy, n(%)					
Yes	75 (13.0)	19 (7.8)	56 (16.8)		0.001*
No	503 (87.0)	226 (92.2)	277 (83.2)		
Pyrexia after delivery, n(%)					
Yes	72 (12.5)	29 (11.8)	43 (12.9)		0.699
No	506 (87.5)	216 (88.2)	290 (87.1)		
Sleep quality during pregnancy, n(%)					
Very good	33 (5.7)	17 (6.9)	16 (6.3)		0.139
Fairly good	321 (55.5)	144 (58.8)	177 (53.2)		
Fairly bad	201 (34.8)	78 (31.8)	123 (36.9)		
Very bad	23 (4.0)	6 (2.4)	17 (5.1)		
Sleep quality after delivery, n(%)					
Very good	62 (10.7)	41 (16.7)	21 (6.3)		< 0.001*
Fairly good	299 (51.7)	145 (59.2)	154 (46.2)		
Fairly bad	195 (33.7)	56 (22.9)	139 (41.7)		
Very bad	22 (3.8)	3 (1.2)	19 (5.7)		
**Medical comorbidities**					
Chronic hypertension, n(%)					
Yes	44 (7.6)	18 (7.3)	26 (7.8)		0.176
No	534 (92.4)	227 (92.7)	307 (92.2)		
Diabetes mellitus, n(%)					
Yes	68 (11.8)	34 (13.9)	34 (10.2)		0.472
No	510 (88.2)	211 (86.1)	299 (89.8)		

Data are presented as or number (percentage) or mean ± standard deviation, unless otherwise indicated. BMI, body mass index; EPDS, Edinburgh postpartum depression scale. * indicates statistical difference

The clinical characteristics of the participants, including antenatal EPDS score, history of abortion, nausea, and vomiting of pregnancy, pyrexia during pregnancy and postpartum, sleep quality during pregnancy and postpartum, and hypertension complicating pregnancy, were analyzed and compared between women with and without PPD. The results indicated that there were significant differences in antenatal EPDS score, pyrexia during pregnancy, and postpartum sleep quality between the participants from the two groups.

### Multiple logistic regression analysis

**Fig. 2:**
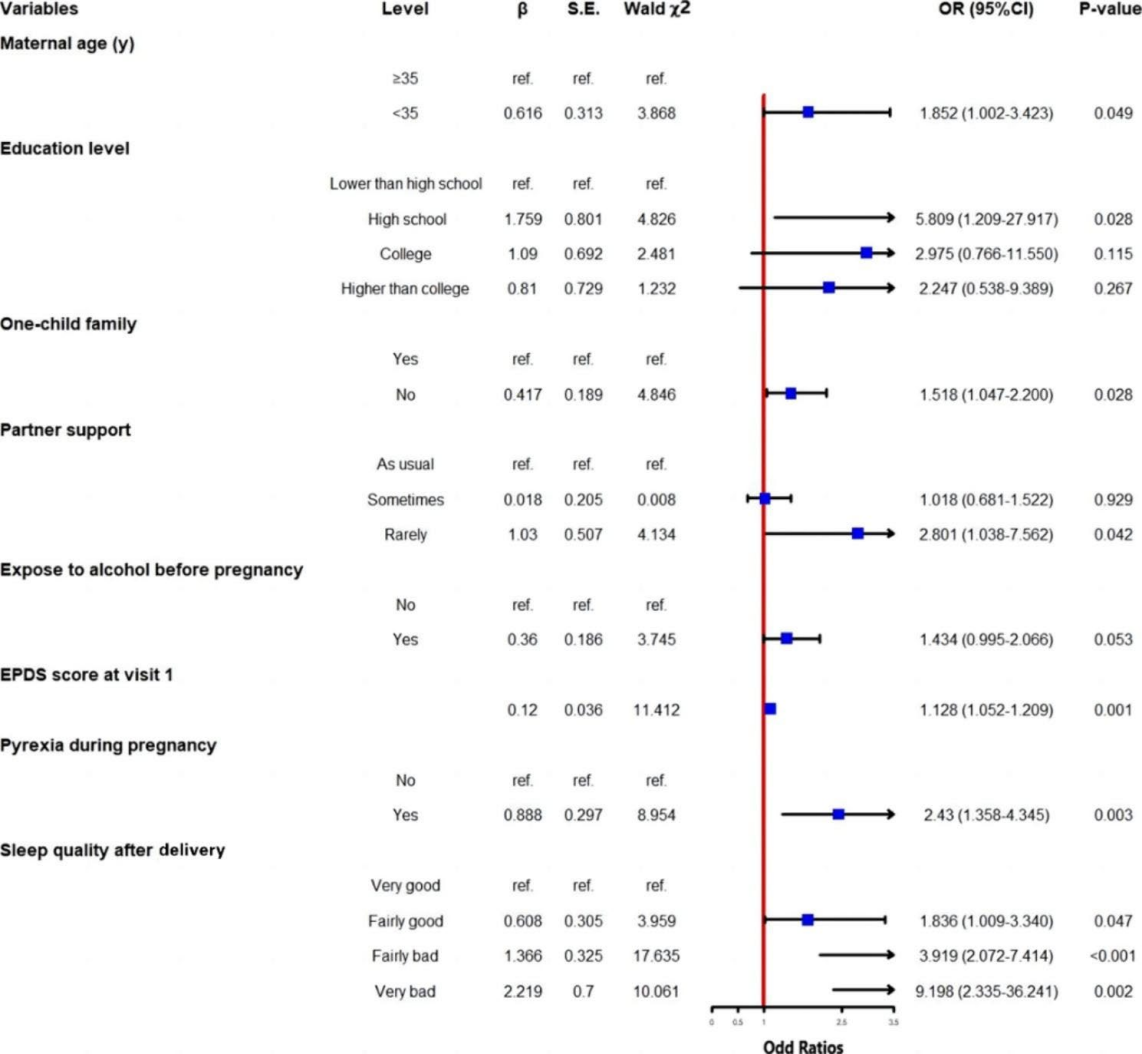
Forest plot of factors associated with postpartum depression

The multiple logistic regression analysis model was employed to identify the predictors of PPD in the participants (Fig. 2). The results showed that age ≤ 35 years old (OR = 1.852; 95%CI: 1.002–3.423), non-one-child families (OR = 1.518; 95%CI: 1.047-2.200), and rare care from spouses during pregnancy (OR = 2.801; 95%CI: 1.038–7.562) were significantly associated with increased risk of PPD in the participants. For clinical characteristics, the antenatal EPDS score (OR = 1.128; 95%CI: 1.052–1.209), pyrexia during pregnancy (OR = 2.43; 95%CI: 1.358–4.345), very good (OR = 1.836; 95%CI: 1.009–3.340), fairly good (OR = 3.919; 95%CI: 2.072–7.414) and fairly poor postpartum sleep quality (compared with those with good postpartum sleep quality) were associated with increased risk of PPD.

**Fig. 3:**
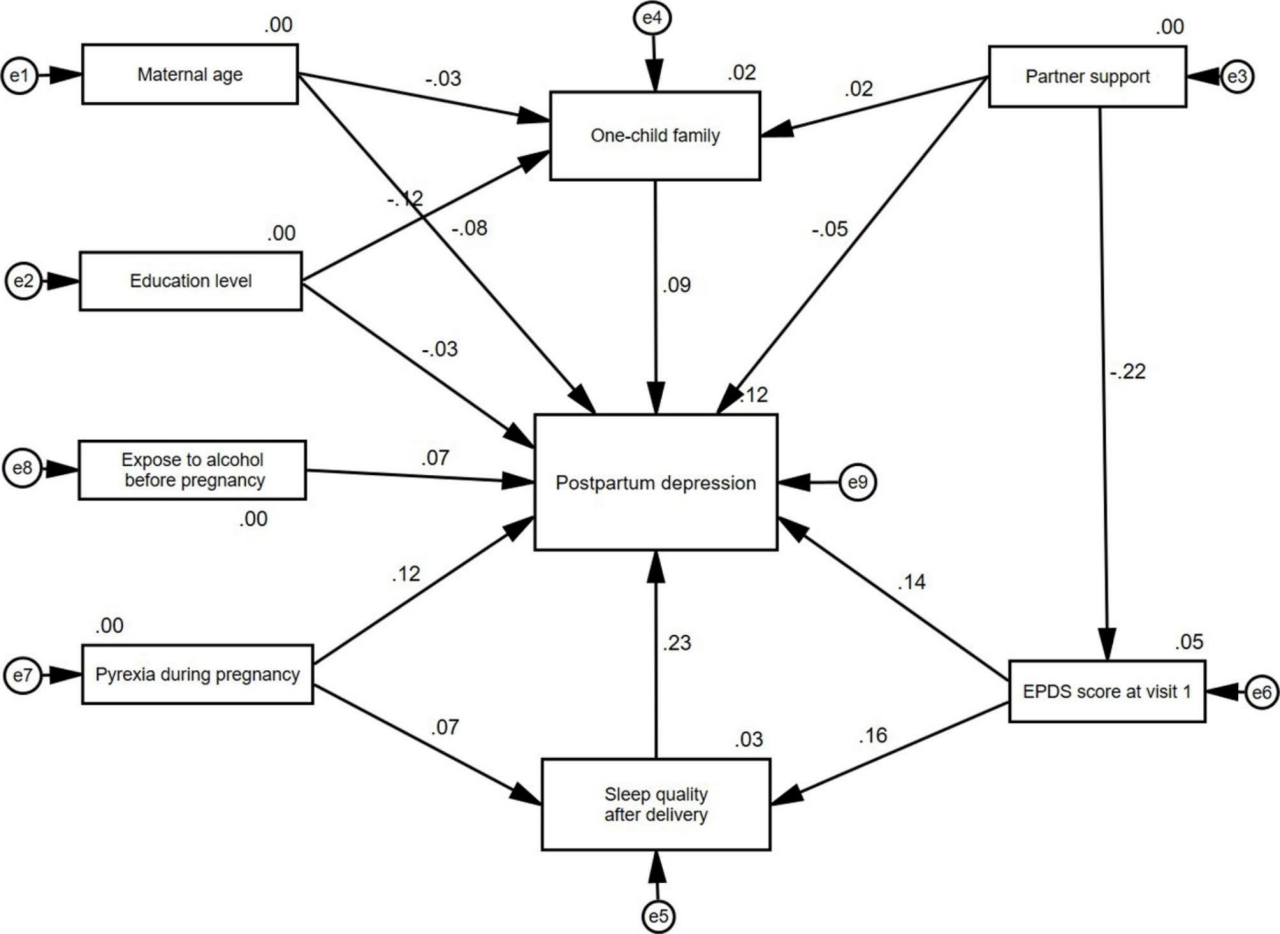
A generalized path analysis model of the risk factors for postnatal depression. All the 9 variables are observational variables: Postpartum depression as the outcome variable and the other 8 variables as independent variables. In the path analysis, the arrow direct point to postpartum depression indicated that the direct effect of doing, while the variables pointed to postpartum depression through other variables represented the indirect effects of doing. One-child family, partner support, EPDS score at visit 1 and Sleep quality after delivery also participated as media variables. In the path analysis model, two variables (sleep quality after delivery, one-child family) had a direct effect to postpartum depression, two variables (partner support, education level) only have indirect effects. On the contrary, the EPDS score at visit 1and pyrexia during pregnancy have both direct and indirect effects. The numbers on the lines indicate the correlation coefficients

### Path analysis model

The fitting results, including the estimated standardized path loadings and interrelationships between the observational variables for the path analysis model, are shown in Fig. 3. The overall model fit was excellent according to the various fit indices (CMIN = 26.740, CMIN/DF = 1.215, *P* = 0.221, RMSEA = 0.019, CFI = 0.962, AGIF = 0.979, ACI = 72.740, BIC = 173.010) (Table 2). Other fit indictors were in Supplementary Table 1. In line with the path analysis, several direct predictors and mediators related to participants’ risk of PPD were identified (Tables 3, 4 and 5). One-child family (p = 0.025), pyrexia during pregnancy (p = 0.003), sleep quality after delivery (p < 0.001), and EPDS score at visit 1 (antenatal EPDS score) (p < 0.001) were all identified as predictors significantly associated with PPD the participants. As shown in Table 4, sleep quality after delivery (standardized β = 0.226, p = 0.004), and one-child family (standardized β = 0.088, p = 0.015) only showed direct effects on the risk of PPD in the participants. In Table 5, Partner support (standardized β=-0.036, p = 0.002) and education level only showed indirect effects (standardized β=-0.011, p = 0.009) on the risk of PPD in the participants. On the contrary, the EPDS score at visit 1 (antenatal EPDS score) has both direct (standardized β = 0.137, p = 0.005) and indirect (standardized β = 0.037, p = 0.004) effects on the risk of PPD in the participants, i.e., EPDS score at visit 1 indirectly influenced PPD via sleep quality after delivery. Pyrexia during pregnancy may also have direct (standardized β = 0.116) and indirect (through sleep quality after delivery, standardized β = 0.017) effects on the risk of PPD, though the indirect effects were not significant (p = 0.051) (Table 5).

**Table 2:** Model fit indices for path analysis of testing a stress process model for postpartum depression

SEM model	CMIN	**Cardinality of freedom ratio (CMIN/DF)**	P-value	CFI	AGFI	RMSEA	**90% confidence interval**		AIC	BIC
							**Low bound**	**Upper bound**		
	26.740	1.215	0.221	0.962	0.979	0.019	0.000	0.042	72.740	173.010

Abbreviations: CMIN: Chi-square; CMIN/DF: Cardinality of freedom ratio (CMIN/DF＜3.00 is acceptable); CFI: comparative fit index (CFI ≥ 0.90 is acceptable, ≥ 0.95 is good ); AGFI: adjusted goodness-of-fit index (AGFI ≥ 0.90 is acceptable, ≥ 0.95 is good ); RMSEA: root mean square error of approximation (RMSEA ≤ 0.08 is recommended); AIC: Akaike’s information criterion; BIC: Bayes information criterion (lower values indicate a better fit, therefore the model with the lowest AIC and BIC is the best fitting model)

**Table 3:** Path coefficients of path analysis for the impact factors of postpartum depression

Paths	Estimates (β)	*SE*	*C.R*-value	*P*-value	Standardized estimates (β)
Partner support → EPDS score at visit 1	-1.091	0.204	-5.361	**< 0.001**	-0.218
Partner support → One-child family	0.014	0.034	0.417	0.676	0.017
Maternal age → One-child family	-0.041	0.067	-0.612	0.541	-0.025
Education level → One-child family	-0.116	0.039	-2.983	**0.003**	-0.123
Pyrexia during pregnancy → Sleep quality after delivery	0.154	0.086	1.781	0.075	0.073
EPDS score at visit 1→ Sleep quality after delivery	0.039	0.010	3.942	**< 0.001**	0.162
Expose to alcohol before pregnancy → Postpartum depression	0.070	0.039	1.824	0.068	0.071
Sleep quality after delivery → Postpartum depression	0.158	0.028	5.720	**< 0.001**	0.226
Pyrexia during pregnancy → Postpartum depression	0.170	0.057	2.958	**0.003**	0.116
One-child family → Postpartum depression	0.090	0.040	2.241	**0.025**	0.088
Maternal age → Postpartum depression	-0.126	0.065	-1.960	**0.050**	-0.076
Education level → Postpartum depression	-0.028	0.038	-0.751	0.452	-0.030
EPDS score at visit 1 → Postpartum depression	0.023	0.007	3.377	**< 0.001**	0.137
Partner support → Postpartum depression	-0.043	0.034	-1.281	0.200	-0.051

**Table 4:** Total and direct standardized effects identified via the path analysis

Paths	Total effects			Direct effects		
	Standardized estimates (β)	95% CI (LB, UB)	P-value	Standardized estimates (β)	95% CI (LB, UB)	P-value
Partner support → EPDS score at visit	-0.218	-0.325, -0.101	**0.004**	-0.218	-0.325, -0.101	**0.004**
Partner support → One-child family	0.017	-0.060, 0.100	0.759	0.017	-0.060, 0.100	0.759
Maternal age → One-child family	-0.025	-0.121, 0.056	0.523	-0.025	-0.121, 0.056	0.523
Education level → One-child family	-0.123	-0.191, -0.043	**0.004**	-0.123	-0.191, -0.043	**0.004**
Pyrexia during pregnancy → Sleep quality after delivery	0.073	-0.007, 0.144	0.076	0.073	-0.007, 0.144	0.076
EPDS score at visit 1→ Sleep quality after delivery	0.162	0.072, 0.24	**0.005**	0.162	0.072, 0.024	**0.005**
Expose to alcohol before pregnancy → Postpartum depression	0.071	-0.016, 0.152	0.086	0.071	-0.016, 0.152	0.086
Sleep quality after delivery → Postpartum depression	0.226	0.147, 0.298	**0.004**	0.226	0.147, 0.298	**0.004**
Pyrexia during pregnancy → Postpartum depression	0.132	0.053, 0.198	**0.005**	0.116	0.038, 0.186	**0.008**
One-child family → Postpartum depression	0.088	0.001, 0.169	**0.015**	0.088	0.011, 0.169	**0.015**
Maternal age → Postpartum depression	-0.079	-0.175, 0.003	0.057	-0.076	-0.167, 0.006	0.071
Education level → Postpartum depression	-0.04	-0.120, 0.040	0.303	-0.03	-0.111, 0.059	0.484
EPDS score at visit 1 → Postpartum depression	0.173	0.101, 0.242	**0.004**	0.137	0.061, 0.208	**0.005**
Partner support → Postpartum depression	-0.087	-0.169, -0.008	**0.03**	-0.051	-0.135, 0.033	0.219

**Table 5:** Indirect standardized effects identified via the path analysis

Paths	Indirect effects		
	Standardized estimates (β)	95% CI (LB, UB)	P-value
Partner support → EPDS score at visit 1 → Sleep quality after delivery	-0.035	-0.066, -0.014	**0.002**
Pyrexia during pregnancy → sleep quality after delivery → Postpartum depression	0.017	0.000, 0.037	**0.051**
Maternal age → One child family → Postpartum depression	-0.002	-0.015, 0.005	0.413
Education level → One child family → Postpartum depression	-0.011	-0.026, -0.002	**0.009**
EPDS score at visit 1 → sleep quality after delivery →Postpartum depression	0.037	0.015, 0.059	**0.004**
Partner support → EPDS score at visit 1 → Postpartum depression	-0.036	-0.068, -0.015	**0.002**

## Discussion

This study aimed to investigate the factors that drive the continuous development of antenatal depression into PPD in a group of women who had experienced antenatal depression in China. The findings from our study suggested that younger age, non-one-child families, rare care from spouses during pregnancy, higher antenatal EPDS score, and pyrexia during pregnancy were significantly associated with increased risk of PPD in the participants, with antenatal EPDS score being the most strongly associated. In particular, we utilized a path analysis model approach to explore the direct and indirect effects of these risk factors on the occurrence of PPD in women. Path analysis, a form of structural equation model, uses “observational variables” and focuses on the correlation mechanisms that may exist among independent variables [23]. It presents a path diagram to show the relationships among the variables, which is clearer than the equations [24]. By specifically decomposing direct, indirect, and total effects, path analysis can lead to a more comprehensive understanding of the relationships among variables. The quantitative expression of regulatory mechanism also makes the analysis more thorough and clear [25]. Therefore, path analysis has been applied to multiple scientific research fields, such as behavioral sciences, social sciences, economics, biology, agriculture, medicine, etc. [18, 26–28]. In addition, since our data were from a cohort, we have also verified the impact of prenatal depression on postpartum depression using longitudinal analyses. The results showed that after adjusting for multiple confounders, prenatal depression remains a strong independent risk factor for postpartum depression, which again demonstrates the important impact of prenatal depression on postpartum depression (Supplementary Table 2).

In this study, 57.6% of the women who had experienced antenatal depression were found to have PPD at 6 weeks after their delivery. Similar to our study, Faisal et al. reported that 49.8% of women with postpartum depression experienced prenatal depression [29]. However, the prevalence of our report was significantly higher than that reported in some previous studies [16, 30], which reported that about 21-25% of the women with antenatal depression would develop PPD at 3 months after childbirth. There are several possible explanations for this discrepancy. Firstly, the cut-off value used to define PPD in our study (EPDS score > 10) was lower than that used in these two studies (EPDS score > 12) [16, 30]. By this criterion, more women would be judged to have PPD in our study. Secondly, there was evidence that notable racial differences and ethnic disparities exist in the risk of PPD among women with different ethnic backgrounds. Green et al. found that, compared with non-Hispanic (NH) whites, Asian women were significantly more likely to report PPD symptoms [31]. Hayes et al. also found that, compared with white women, Chinese women had higher odds of self-reported PPD symptoms (adjusted odds ratio [AOR] = 2.0; 95% CI: 1.5–2.7) [32]. As the participants of our study were Chinese women, a higher prevalence of PPD can be expected. Moreover, the following-up time of our study (6 weeks) was shorter than that of the study conducted by Redshaw et al. (3 months) [16]. Aasheim et al. found that the prevalence of psychological distress in women after childbirth showed a U-shaped pattern over time up to 18 months, with the lowest point at 6 months [33]. Therefore, the prevalence of PPD may be higher among women at 6 weeks than that of women at 3 months after childbirth. Taken together, these results suggested that the prevalence of PPD in women after childbirth may be altered by the diagnostic criterion, race/ethnic, and following-up time.

The results from logistic regression analysis and structural equation model described the predictors of and their cause mechanisms on the occurrence of PPD. Maternal educational level, partner support, and one-child family may reflect the social support of the women, which were found to be significantly associated with the occurrence of PPD. This finding was consistent with those of the study conducted by Yamada et al., which found that mothers with low social support from partner or others had significantly higher odds to have PPD, in comparison with mothers who have high social support [34]. Desta et al. also found that mothers with inadequate social support (OR = 5.46,95%CI: 3.94, 7.56) or suffer from intimate partner violence (OR = 6.27,95%CI: 4.83, 8.13) had a significantly higher risk of PPD relative to those who had adequate social support. These findings highlight the importance of social support in the development of PPD. Studies such as Alessandra et al. believe that domestic violence is a risk factor for postpartum depression [35]. Nonetheless, studies regarding intimate partner violence (IPV) in China are far less than other countries [36]. A possible explanation is that due to traditional Chinese culture, family affairs are usually kept private and women are reluctant to report IPV. As a consequence, although there have been some studies on domestic violence in recent years in China, the rates seemed to be underreported. For example, in one study for risk factors of antenatal depression, only one domestic violence was reported [37]. Therefore, in our study, such information is lacking. In future research, we will try to incorporate such information and evaluate the impact of domestic violence on postpartum depression.

The clinical characteristics, including pyrexia during pregnancy, the antenatal EPDS score, and sleep quality after childbirth were found to be major contributors to the risk of PPD in the participants of our study. Pyrexia during pregnancy, an indicator of the infectious status [38], has been demonstrated to be an important risk factor for both the physical and mental health of pregnant women [39]. This was supported by findings from previous studies, which found that persistent fever or dengue fever during pregnancy were key determinants of depressive symptoms among postpartum mothers [39, 40]. The positive and significant association between antenatal EPDS score and risk of PPD in our study suggested the continuity and stability of depressive symptoms from the antenatal to the postpartum stage. As antenatal depression is one of the strongest predictors of PPD [41], this finding highlight the importance of the prevention and early intervention of depression in pregnant women [42, 43]. Sleep disturbance, which is caused by the factors such as alterations of the hormones, physical discomforts, the presence of restless legs syndrome (RLS), and feeding and caretaking behaviors [44], is a common problem for mothers during the postpartum period [45]. Our study found that sleep quality had dose-response effects on the odds of PPD, illustrating the central role of poor sleep quality in the development of PPD. These results were in line with those of previous studies, which found that sleep disturbance was significantly associated with an increase in the odds of PPD [46, 47]. However, we only found the impact of postpartum sleep quality on postnatal depression, but did not find the impact of prenatal sleep quality, which is inconsistent with previous studies [48]. We think that the impact of sleep during pregnancy on postpartum depression is not absolute, all these findings revealed that PPD was a complex disorder that results from an intricate interplay among various social, physical, and psychological factors.

The results from the structural equation model indicated that, besides their direct effects, pyrexia during pregnancy and antenatal depression had indirect effects on the occurrence of PPD through their impacts on the sleep quality after delivery. This finding was in agreement with those obtained by Wang et al., which found that poor sleep quality mediated the relationship between antepartum depressive symptoms and postpartum depressive symptoms [49]. Though lack of direct evidence, this result was supported by the data from adult patients with familial Mediterranean fever, which demonstrated that fever may increase inflammatory marker levels and lead to poor sleep quality and depression symptoms [50]. Similar associations were observed for these social support-related factors, including maternal age, partner support, and educational level, though some were not statistically significant. These findings seem to be consistent with other research which found social support had a direct impact on depressive symptoms and mediated the effect of stress on depressive symptoms during pregnancy [51]. These results described the causal mechanisms linking socio-demographic and clinical characteristics to PPD among women who experienced antenatal depression.

### Strength, limitation, and public health implications

To our knowledge, this is the first study to explore the risk factors and their causal mechanisms driving the continuous development of antenatal depression into PPD in women from an urban region in China. As we used a nested sample from a cohort study and a path analysis frame to analyze the data, a conceptual framework on the relationship between risk factors for PPD can be developed with a relatively low attrition rate. This approach allowed us to simultaneously assess the direct and indirect associations between potential risk factors and PPD and describe the possible explore potential causal mechanisms.

This study has several limitations. Firstly, though a nested case-control design was employed and the associations between predictors and PPD were demonstrated in this study, the causality cannot be inferred. Secondly, we have only focused on the impact of family support on depression but without paying attention to the negative impact of domestic violence, this would be of value to research in future studies in China. Moreover, although we enrolled the participants from one of the major obstetrics and gynecology hospitals in Hangzhou, the findings from this study cannot be generalized nationally. Last but not least, the proportion of primipara in our population is relatively high. With the changes in fertility policies, we will focus on the impact of the number of pregnancies and births on postpartum depression in the next few years.

The study has implications for preventing and controlling PDD in Chinese women. The identified predictors played an important role in the risk of PPD in our participants. Prevention and early treatment of antepartum depressive symptoms, infection, and sleep disturbance in women may help to eliminate the risk of PPD in this population. Similarly, since social and partner support were found to have mediating and buffer effects on the risk of PPD, promoting activities that persevere such social capitals are needed to maintain the psychological and mental well-being of mothers.

## Conclusion

In summary, the results from our study indicated that more than 50% of the women who experienced antepartum depression would subsequently develop PPD. Based on our findings, severe antenatal depressive symptoms, pyrexia during pregnancy and poor sleep quality after delivery are the main risk factors for PPD. Social and partner support was found to buffer or reduce the risk of PPD. The insights gained from this study may be of assistance in comprehensively understanding the etiology of PPD in women who experienced antenatal depression.

## Electronic supplementary material

Below is the link to the electronic supplementary material.

Supplementary Material 1

## Data Availability

The datasets generated and/or analyzed during the current study are not publicly available due to ethic issues involving participant’s data and privacy but are available from the corresponding author on reasonable request.
