# Supplementary material for: Risk factors for the development of postpartum depression in individuals who screened positive for antenatal depression
Source: BMC Psychiatry. 2023 Aug 1;23:557. doi: 10.1186/s12888-023-05030-1 (PMC10394808; doi:10.1186/s12888-023-05030-1)
Supplement: Supplementary file 1 — Supplementary Material 1 [file 12888_2023_5030_MOESM1_ESM.docx]

Table 1. Fitting of each fit index of structural equation model

| **Fit indicator** | **Result** | **Judgement** | **Fit criteria** |
| --- | --- | --- | --- |
| χ^2^/*df* (CMIN/DF) | 1.215 | Good | <5 is acceptable，<3 is good |
| Goodness-of-fit index (GFI) | 0.990 | Good | >0.80 is acceptable，>0.90 is good |
| Adjusted goodness-of-fit index (AGFI) | 0.979 | Better | >0.90 is good，>0.95 is better |
| Root mean square residual (RMR) | 0.014 | Better | <0.10 is good，<0.05 is better |
| Root mean squared error of approximation (RMSEA) | 0.019 | Better | <0.10 is good，<0.05 is good |
| Normed fit index (NFI) | 0.833 | Acceptable | >0.80 is acceptable，>0.90 is good |
| Incremental fit index (IFI) | 0.966 | Good | >0.80 is acceptable，>0.90 is good |
| Tucker–Lewis index (TLI) | 0.938 | Good | >0.80 is acceptable，>0.90 is good |
| Comparative fit index (CFI) | 0.962 | Good | >0.80 is acceptable，>0.90 is good |

**Abbreviations**: Absolute Goodness-of-Fit Indices: CMIN/DF, GFI, AGFI, RMR, RMSEA; Relative Goodness-of-Fit Indices: NFI, IFI, TLI, CFI.

Table 2. Cox regression model for the effect of postpartum depression

| Variable | Model 1 | | Model 2 | | Model 3 | |
| --- | --- | --- | --- | --- | --- | --- |
|  | *HR* (95%CI) | *P*-value | *HR* (95%CI) | *P*-value | *HR* (95%CI) | *P*-value |
| Antenatal depression | 2.632(2.270-3.052 | <0.001 | 2.585(2.227-3.000) | <0.001 | 2.626(2.262-3.049) | <0.001 |

**Abbreviations**: Model 1 was non-adjusted; Model 2 was adjusted for age (years), education level, household yearly income (ten thousand CNY); Model 3 was further adjusted for BMI, one-child family, Primipara.
